# A new genus and species of Staphylininae rove beetle from the Peruvian Amazon (Coleoptera, Staphylinidae)

**DOI:** 10.3897/zookeys.904.48592

**Published:** 2020-01-16

**Authors:** Josh Jenkins Shaw, Igor Orlov, Alexey Solodovnikov

**Affiliations:** 1 Key Laboratory of Zoological Systematics and Evolution, Institute of Zoology, Chinese Academy of Sciences, Beijing, 100101, China Institute of Zoology, Chinese Academy of Sciences Beijing China; 2 Natural History Museum of Denmark, Zoological Museum, Universitetsparken 15, Copenhagen 2100, Denmark Natural History Museum of Denmark Copenhagen Denmark

**Keywords:** Hyptiomini, Neotropical Region, Peru, South America, Tanygnathinini, taxonomy

## Abstract

A new monotypic genus of Staphylininae Latreille, 1802 tribe *incertae sedis* is proposed based on *Amazonothops aslaki***gen. et sp. nov.** from the Peruvian Amazon. Descriptions and illustrations of the new genus and species are provided. Its systematic placement and phylogenetic significance are discussed.

## Introduction

**Figure 1.**
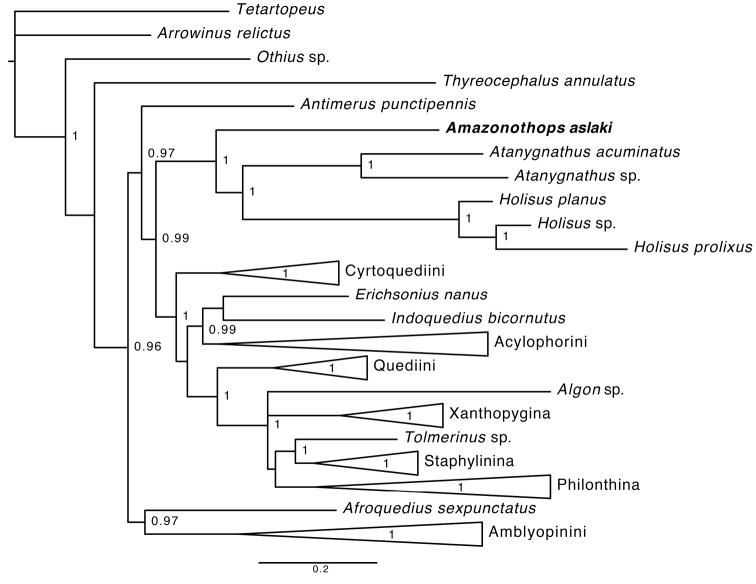
Majority-rule consensus tree from the Bayesian analysis of Jenkins Shaw et al. (2019), showing the position of *Amazonothops aslaki*. Major clades have been collapsed. Posterior probabilities of 0.96 and higher are provided.

During a field trip to the Amazonian region of Peru, members of the Natural History Museum of Denmark Coleoptera section collected several conspecific specimens of a small rove beetle which strongly resembled the widespread, polyphyletic genus *Heterothops* Stephens, 1829 from the tribe Amblyopinini (Jenkins Shaw et al. 2019). One of these specimens was included in the molecular phylogenetic analysis of Jenkins Shaw et al. (2019). Whilst that study primarily sought to investigate the phylogeny of the tribe Amblyopinini, it also included a broad sample of representative Staphylininae and other subfamilies of Staphylinidae as outgroups. The results of that phylogeny recovered the Amazonian taxon as sister to the tribes Tanygnathinini and Hyptiomini with strong support, far away from *Heterothops* and even Amblyopinini as a whole (Fig. 1). Further morphological study revealed numerous unusual characters that confirmed it indeed did not belong to the genus *Heterothops* or even the tribe Amblyopinini. At the same, it was morphologically distinct from either Tanygnathinini or Hyptiomini. Without hesitation we describe *Amazonothops aslaki* gen. et sp. nov. Even though we place this new genus as *incertae sedis* in the subfamily Staphylininae pending further inquiry, we provide comparisons and discussion to explain why *Amazonothops* is an important discovery for understanding evolution of the morphologically heterogeneous and still rather enigmatic clade it belongs to.

## Material and methods

Specimens studied are deposited in the following institutions:

**NHMD**Natural History Museum of Denmark, University of Copenhagen, Denmark (Curator Alexey Solodovnikov)

**SEMC**Snow Entomological Museum Collection, University of Kansas, USA (Collection Manager Zack Falin)

One specimen was prepared for scanning electron microscopy (SEM) by initial immersion in soapy water, followed by 1 hour in 10% KOH, resting overnight in 70% alcohol, then successive immersion in 96% alcohol for 15 minutes, in 99.9% alcohol for 15 minutes and in acetone for 15 minutes. SEM photographs were taken using a JEOL JSM-6335F SEM at the NHMD. One specimen was slide mounted using the method described in Hanley and Ashe (2003): slide photographs were taken using a Canon EOS 6D DSLR (Canon Inc.) digital camera mounted on a Zeiss Axioskop 50 via a LM Digital SLR Universal Adapter. Images were captured from multiple focal planes with the help of Canon EOS utility 3.4.30.0 software (Canon Inc.), combined with Zerene Stacker (Zerene Systems LLC, Richland, USA) software and edited with Adobe Photoshop CS6 and Adobe Illustrator CS6 (Adobe Systems, San Jose, CA, U.S.A) afterwards. The map in Fig. 6 was generated using SimpleMappr (Shorthouse 2010). Holotype or paratype labels have been added to all type specimens, respectively. Measurements were taken using ImageJ and calibrated based on the scale bar in the images. Measurements were based on a single specimen as all individuals studied exhibited very little or no variation in size or proportions.

The following measurements were taken (all in mm):

**HW** Head width, at widest point

**PL** Pronotum length, at middle

**PW** Pronotum width, at widest point

**FB** Forebody length, from anterior edge of frons to posterior end of elytral suture

**EW** Elytral width, at widest point

**TL** Total length, from anterior edge of frons to apex of abdominal segment IX

## Results

### Family Staphylinidae Latreille, 1802

#### Subfamily Staphylininae Latreille, 1802

**Tribe *incertae sedis***

##### Genus Amazonothops gen. nov.

Taxon classificationAnimaliaColeopteraStaphylinidae

B7E8788E-11A8-547F-9EC0-D8CF33938885

http://zoobank.org/36850046-A545-4133-8ECB-8D6357BFAC53

Figs 2 , 3 , 4 , 5

###### Type species.

*Amazonothops aslaki* gen. et sp. nov.

###### Diagnosis.

From all other genera of Staphylininae the new genus can be recognized based on the following characters: antennomere 2 1.6× wider than antennomere 3; antennomere 11 3× longer than antennomere 10; penultimate segment of maxillary palpi large, covered in short setae, approximately 2× the length of apical segment; apical segment of maxillary and labial palpi aciculate. Head with ‘infraorbital ridges’ straight, extended to base of mandibles; postgenal ridge absent; frontoclypeal suture present; mesoventrite with transverse ridge present (incomplete medially); mesotrochanter and first mesotarsomere of males with black combs; tarsal formula 5-5-5; empodial setae long and parallel-sided; apical edge of sternites III to VI with randomly distributed acute projections; tergites VII and VIII with broader, foliose setae in addition to the usual acuminate, simple setae; apparent fusion of tergite X to lateral tergal sclerites in males.

###### Differential diagnosis.

The differential diagnosis is based on the recovered phylogenetic position of *Amazonothops* and its strong resemblance to the Amblyopinini genus *Heterothops*. *Amazonothops* differs from *Atanygnathus* Jacobson, 1909 (Tanygnathinini) in the number of tarsal segments (5-4-4 in *Atanygnathus*); short genae and normal shape of the apical labial and maxillary palpomere (extremely elongate and distinctly converging to apex in *Atanygnathus*); absence of dorsal setae on the apical tarsomere (present in *Atanygnathus*). It should be noted that some species of *Atanygnathus* have combs on the profemora (Adam Brunke, personal communication). *Amazonothops* differs from species of *Holisus* Erichson, 1839 (Hyptiomini) in the pronotal hypomeron strongly inflexed, not visible in lateral view and without longitudinal middle carina (visible in lateral view and with middle carina in *Holisus*), presence of empodial setae (absent in *Holisus*) and general appearance and habitus (*Holisus* is distinctly dorso-ventrally flattened with coarse punctation). *Amazonothops* differs from the genus *Natalignathus* Solodovnikov, 2005, a hitherto unrecognized possible member of the same clade (see Discussion below) in the smaller body, short genae and much lesser elongate mouthparts, absence of dorsal setae on the apical tarsomere (present in *Natalignathus*) and presence of the combs. *Amazonothops* differs from *Heterothops* and other genera of Amblyopinini in presence of the frontoclypeal suture; antennomere 3 distinctly smaller than antennomeres 2 and 4; mesosternum with transverse ridge present (incomplete medially); apical edge of sternites III to VI with randomly distributed acute projections; tergites VII and VIII with broader, foliose setae in addition to the usual acuminate, simple setae; and apparent fusion of tergite X to lateral sclerites in males.

###### Description.

**Figure 2.**
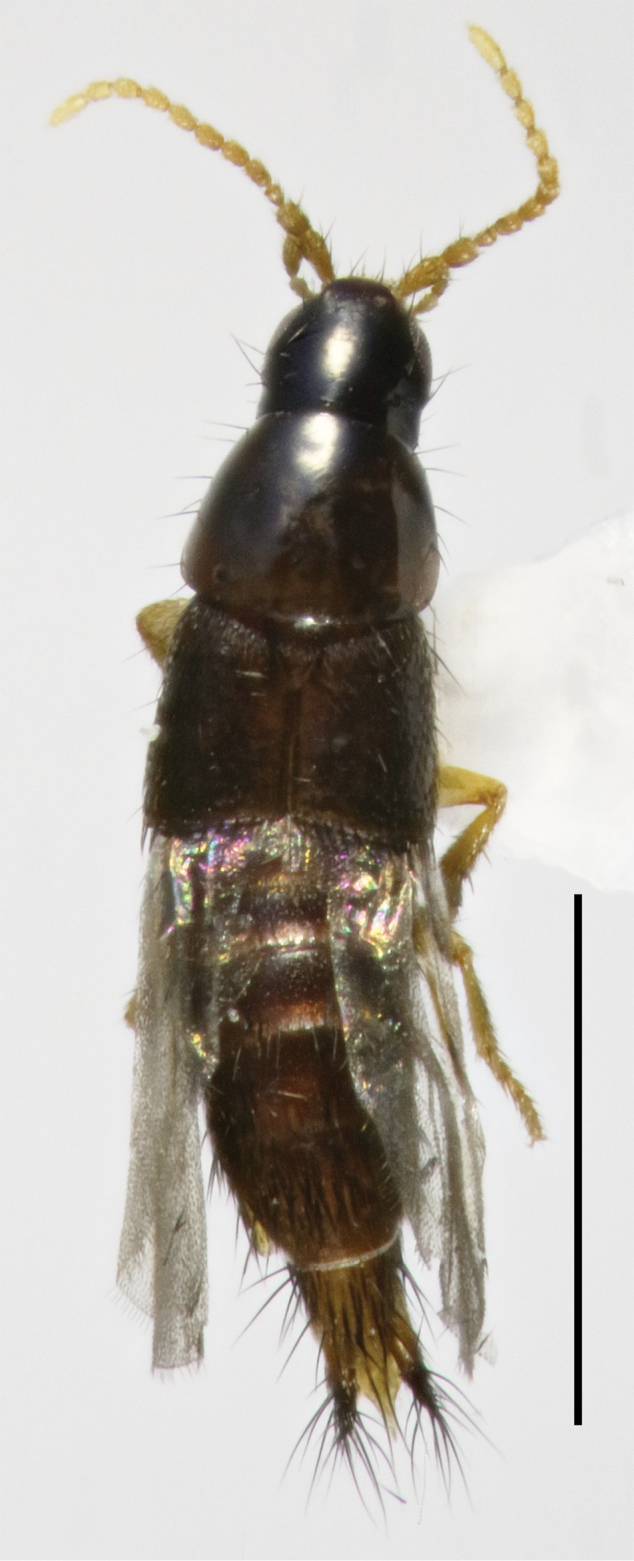
Habitus of *Amazonothops aslaki* gen. et sp. nov. Scale bar: 1 mm.

Habitus as in Fig. 2. Body dark brown-black; antennae and legs yellowish. Measurements (all in mm): HW = 0.31; PL = 0.38; PW = 0.48; FB = 1.00; EW = 0.55; TL = 2.19.

**Figure 3.**
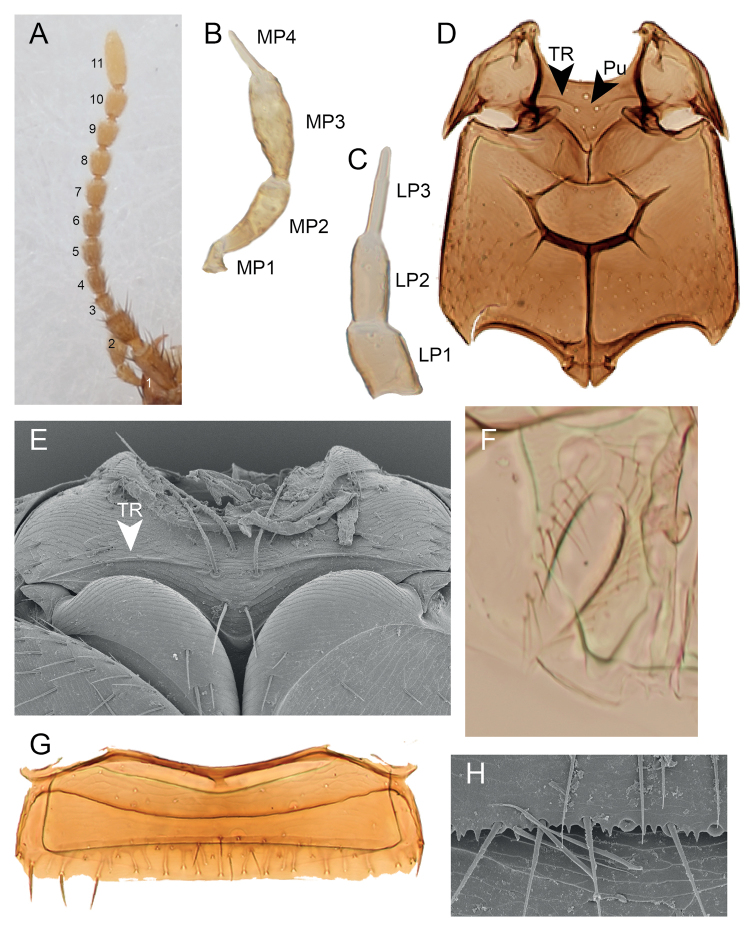
Morphology of *Amazonothops aslaki* gen. et sp. nov. **A** antennae **B** maxillary palpi **C** labial palpi **D** meso- and metathorax **E** mesothorax **F** protergal gland **G** tergite II **H** sternite III (apical edge). LP, Labial palpomere; MP, Maxillary palpomere; Pu, punctures; TR, Transverse ridge.

***Head.*** Dorsal surface with weak transverse microsculpture. Neck indistinct; nuchal ridge absent dorsally, present laterally, extended as ‘infraorbital ridge’ towards base of mandibles. Frontoclypeal suture present. Frontoclypeal puncture present (Brunke et al. 2019: fig. 1). Anterior and posterior frontal punctures present (Brunke et al. 2019: fig. 1). Single basal puncture present (Brunke et al. 2019: fig. 1). Eyes occupying two thirds of the side of head; temples indistinct. Antennae (Fig. 3A) inserted close to margin of eye. Antennomeres 1 to 3 setiferous; 4 to 11 setiferous and with tomentose pubescence. Antennomere 2 1.6× the width of antennomere 3; antennomere 3 half the size of antennomere 2; antennomere 11 elongate, three times as long as antennomere 10. Gula widest in anterior half; gular sutures separated along entire length; postgenal ridge absent. Maxillary palpi four-segmented (Fig. 3B; apical segment acicular, about a third of the length of the penultimate palpomere; penultimate segment widest at middle; covered with setae. Labial palpi three-segmented (Fig. 3C); apical segmented acicular. Labrum transverse, complete, without emargination. Mandibles simple, crossing in resting position, without large teeth.

***Prothorax.*** Pronotum widest in posterior third. Dorsal surface with weak transverse microsculpture and two pairs of punctures in dorsal series (one puncture near posterior margin and one distad of that); hypomeron strongly inflexed (not visible in lateral view). Basisternum with weak longitudinal ridge in posterior half, without punctures or setae. Post-coxal process absent.

Scutellum with anterior transverse ridge only, impunctate, glabrous. Elytra widest posteriorly. Hind wings fully developed, posterior edge with fringe of setae; veins CuA and MP4 fused; vein MP3 present. Mesoventrite (Fig. 3D) with five large punctures medially (Fig. 3D; Pu), with rounded ventral process and with transverse ridge, incomplete medially (Fig. 3E).

**Figure 4.**
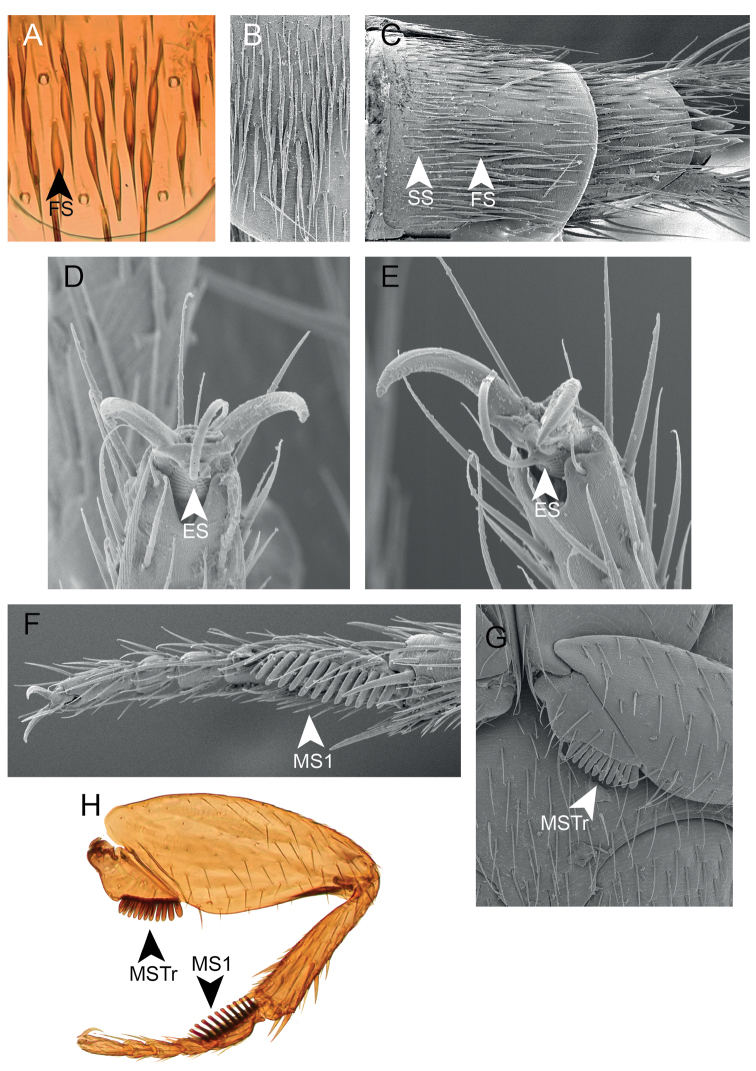
Morphology of *Amazonothops aslaki* gen. et sp. nov. **A** tergite VII **B** tergite VII **C** abdominal apex (tergites VII and VIII) **D** empodium of mesotarsi **E** empodium of metatarsi **F** mesotarsi (male) **G** mesotrochanter (male) **H** mesoleg (male). ES, Empodial setae; FS, Foliose setae; MS, Mesotarsomere; MSTr, Mesotrochanter; SS, Simple setae.

***Abdomen.*** Protergal glands elongate, fringed by setae (Fig. 3F). Tergites with anterior transverse carina only. Tergite II as in Fig. 3G. Sternite III with evenly curved transverse carina, slightly projected medially. Tergite VII with white fringe along posterior edge. Apical edge of sternites III to VI with randomly distributed acute projections (Fig. 3H). Tergites VII and VIII with broader, foliose setae in addition to the usual acuminate, simple setae (Fig. 4A–C).

***Legs.*** Tarsal formula 5-5-5. Both sexes with protarsomeres 1 to 4 transverse. All tarsal empodia with long, parallel-sided setae (Fig. 4D, E).

***Male.*** Protarsomeres 1 to 4 with white adhesive setae ventrally. First mesotarsomere with black comb comprising 11–14 articles (Fig. 4F, H). Mesotrochanter with black comb comprising 7–11 articles (Fig. 4G, H). The number of articles within each comb varies between individuals.

***Female.*** Protarsomeres 1 to 4 only with usual setae ventrally, white adhesive setae absent. No combs.

###### Distribution.

Based on the specimens studied here, the new genus is restricted to the lowland areas of the Amazonian basin of Peru.

###### Bionomics.

Based on the available label data, the genus occurs in forested areas (100–420 m elevation) and has so far only been collected by flight intercept traps.

###### Etymology.

The genus name is a combination of ‘Amazon’ and the genus name *Heterothops*, which the new genus strongly resembles superficially.

###### Remarks.

The new genus is certainly morphologically distinct among other members of the subfamily Staphylininae and family Staphylinidae. Noteworthy is the secondary sexual dimorphism exhibited by *Amazonothops*. In males, the mesotrochanter and first mesotarsomere have distinct black combs which are completely lacking in females (Fig. 4F, G, H). The function, if any, of the combs remains unknown but very similar combs are commonly found within Amblyopinini, e.g., recently illustrated for *Myotyphlus* Fauvel, 1883 (Solodovnikov and Jenkins Shaw 2017).

##### Amazonothops aslaki sp. nov.

Taxon classificationAnimaliaColeopteraStaphylinidae

76FECFFB-9BCB-51C4-9D45-C4AA5982C41E

http://zoobank.org/1B5BA8C2-CD02-428D-A21B-FFD807C74724

Figs 2 , 3 , 4 , 5

###### Material examined.

***Holotype.*** Male ‘PERU: Amazonia, Loreto region, Requena Province, 3km E of Jenaro Herrera, 100–200 m, 4°53.914'S, 73°38.689'W, 24–28.VIII.2017, rainforest, FIT close to logs, A. Hansen, D. Zyla, M. Chani-Posse PER17-11i’ (NHMD). ***Paratypes.*** 2 males (of them 1 DNA vouchered), 1 female, same locality but 4°53.210'S, 73°38.921'W, 7–10.IX.2017, rainforest, FIT near wetland/*Mauritia flexuosa* L.f. palms, leg. A. Hansen, J. Kypke, A. Solodovnikov (PER 17-22f) (NHMD). Male (coated for SEM and mounted on two stubs), same data, but FIT near creek (PER17-22l) (NHMD). 1 male, disarticulated and slide mounted, ‘PERU: Dept. Madre de Dios: Pantiacolla Lodge, Alto Madre de Dios R. 12°39.3'S, 71°13.9'W 420m 14-19-XI-2007 D. Brzoska ex. flight intercept trap PER1B07 004 / SEMC0874476 KUNHM-ENT’ (SEMC).

###### Description.

**Figure 5.**
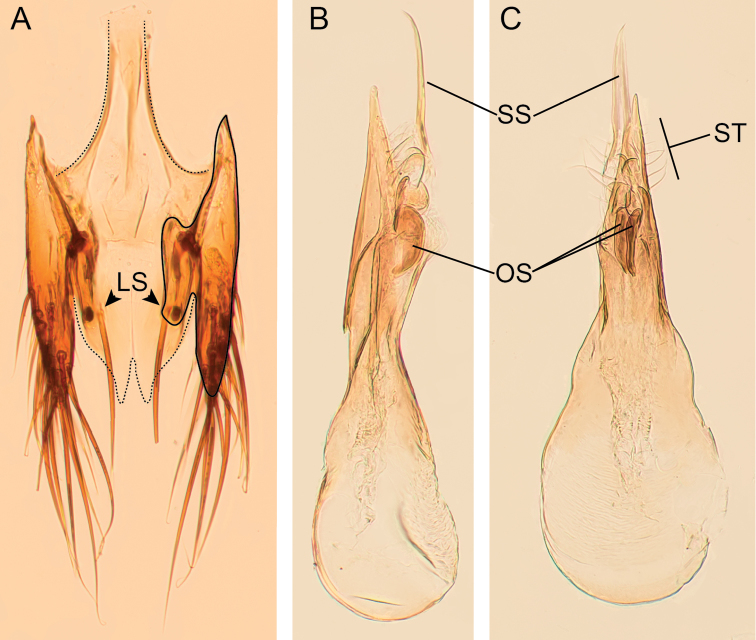
Morphology of *Amazonothops aslaki* gen. et sp. nov. **A** male terminalia (abdominal segments IX, X) **B** aedeagus in lateral view **C** aedeagus in antiparameral view (slightly lateral). LS, Long setae. OS, Oblong sclerite. SS, Slender sclerite. ST, setae.

In addition to characters in the genus description, the species is characterized by the following primary and secondary male sexual characters. Sternite VIII without apical incision. Tergite X apparently fused to internal face of lateral sclerites (Fig. 5A, solid line), with two large setae situated at apical third of length (Fig. 5A; LS). Sternite IX emarginate apically, with symmetrical basal stem (Fig. 5A, dashed line). Lateral tergal sclerites and tergite X of approximately equal length. Aedeagus (Fig. 5B, C); paramere longer than and closely attached to, median lobe (Fig. 5B); lateral apical area of paramere with setae of varying length (Fig. 5C; ST); internal sac consisting of a pair of heavily sclerotized oblong-shaped sclerites (Fig. 5C; OS) and pair of weakly sclerotised longer, more slender sclerites extruding from apical area of median lobe under pressure (Fig. 5C, SS).

###### Distribution.

**Figure 6.**
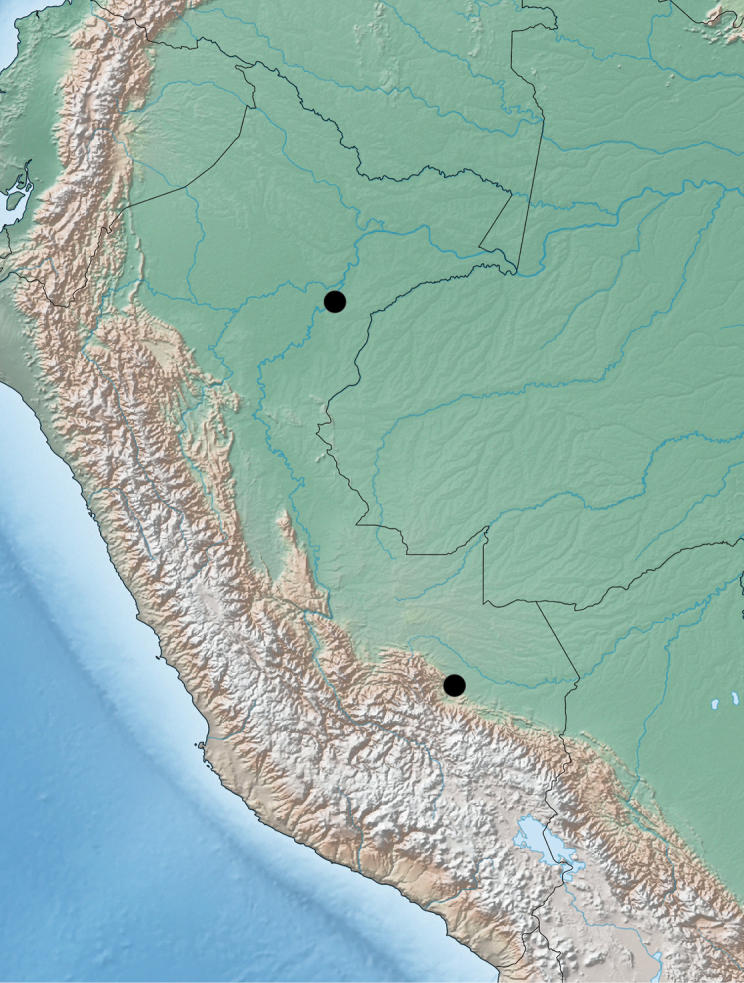
Known distribution of *Amazonothops aslaki* gen. et sp. nov. in Peru.

Based on the specimens studied here, the new genus is restricted to the lowland areas of the Amazonian basin of Peru (Fig. 6). As a fully winged species known only from six specimens from two areas approximately 1000 kilometres apart from each other, it seems to be a widespread species, perhaps much more than it appears now.

###### Bionomics.

Same as above.

###### Etymology.

The species is named in honour of Aslak Kappel Hansen (NHMD) who was one of the collectors of this new genus and who brought our attention to its unusual nature compared to *Heterothops*.

###### Comments.

It is notable that a species of *Heterothops* (Amblyopinini) was obtained during the same collecting event as one of the *Amazonothops* specimens. This shows that these two similar looking, yet phylogenetically distant (Fig. 1) taxa co-occur, probably in similar or the same microhabitats in the Amazon basin.

## Discussion

The molecular phylogeny of Jenkins Shaw et al. (2019) placed *Amazonothops* sister to a peculiar clade formed by the tribes Tanygnathinini and Hyptiomini with maximal support (Fig. 1). As Jenkins Shaw et al. (2019) noted, the recovery of *Amazonothops* in a clade with Tanygnathinini and Hyptiomini also makes the somewhat similar South African endemic genus *Natalignathus* relevant when considering the sister relationships of *Amazonothops*. *Natalignathus* was originally placed in Tanygnathinini (Solodovnikov 2005) and later moved to Amblyopinini (then Amblyopinina) by Chatzimanolis et al. (2010). Although the phylogenetic study by Chatzimanolis et al. (2010) was molecular, *Natalignathus* was reclassified based on a morphological character assessment and not included in the analysis as DNA-grade material was not available. Besides, previous morphological studies considered it to be sister to *Atanygnathus*, itself considered a highly autapomorphic lineage nested within Amblyopinini, a pattern repeatedly revealed in morphology-based phylogenetic analyses since Solodovnikov (2006). Tanygnathinini were not sunk in synonymy to Amblyopinini by these authors because of their very clear morphological diagnosis and a lack of knowledge of the internal phylogeny of Amblyopinini. Chatzimanolis et al. (2010) were the first to link *Holisus* and *Atanygnathus* in an isolated clade within Staphylininae, though they are so different morphologically that this was suspected to be an artefact. However, this sister group relationship between Tanygnathinini and Hyptiomini, persisted through several later molecular (Brunke et al. 2016; Żyła and Solodovnikov 2019; Jenkins Shaw et al. 2019) and total evidence (Chani-Posse et al. 2018) phylogenetic studies with better and more diverse taxon and gene sampling. Unfortunately, *Natalignathus* remains unsampled for molecular phylogeny due to a lack of DNA-grade material. In view of the continued corroboration of the Tanygnathinini + Hyptiomini clade, and now with *Amazonothops* discovered as a sister group to that, it seems plausible that *Natalignathus* should belong to the Tanygnathinini + Hyptiomini clade as well, probably as a sister to *Atanygnathus* as was initially noted at the time of its description (Solodovnikov 2005). Even though peculiar, the morphology of *Amazonothops* seems less derived than either *Holisus* or *Atanygnathus*, showing some resemblance, and sharing some characters with, *Natalignathus*. Like *Natalignathus*, *Amazonothops* resembles and shares some characters with the early-diverging and plesiomorphy-rich ‘Quediine-looking’ lineages of Staphylininae, much more so than the derived *Atanygnathus* or *Holisus*. *Amazonothops* is clearly an important taxon for understanding of the origin the entire Tanygnathinini + Hyptiomini clade. Once the molecular data from *Natalignathus* is obtained, both it and *Amazonothops* should be included in a total evidence phylogenetic analysis to explore the early evolution of Staphylininae. Discovery of *Amazonothops* further highlights the rich and unknown biodiversity of the Peruvian Amazon and suggests a high probability that more species of this genus, or even new genera of this lineage maybe discovered there in the future.

With the recently updated classification of the subfamily Staphylininae (Żyła and Solodovnikov 2019) and the recovered phylogenetic position of *Amazonothops* (Jenkins Shaw et al. 2019), it is plausible that *Amazonothops* should be placed in its own tribe. Here we refrain from doing so pending further molecular and morphological exploration of the (*Amazonothops* (*Atanygnathus* + *Holisus*)) clade with broad outgroup sampling that would also include sampling many more ingroup species for the latter two sizeable genera, and revisiting *Natalignathus*.

## Supplementary Material

XML Treatment for Amazonothops

XML Treatment for Amazonothops aslaki
