# Corrigendum: A new genus and species of Staphylininae rove beetle from the Peruvian Amazon (Coleoptera, Staphylinidae). https://doi.org/10.3897/zookeys.904.48592

**DOI:** 10.3897/zookeys.912.50592

**Published:** 2020-02-17

**Authors:** Josh Jenkins Shaw, Igor Orlov, Alexey Solodovnikov

**Affiliations:** 1 Institute of Zoology, Chinese Academy of Sciences, Beijing, China Institute of Zoology, Chinese Academy of Sciences Beijing China; 2 Natural History Museum of Denmark, Copenhagen, Denmark Natural History Museum of Denmark Copenhagen Denmark

## Abstract

In a recent paper (Jenkins Shaw et al. 2020) we stated that the holotype of Amazonothops aslaki would be deposited in the Natural History Museum of Denmark (NHMD). According to the conditions of the permit RDG 0328-2017-SERFOR-DGGSPFFS/RDG 356-2017-SERFOR-DGGSPFFS, the holotype and one paratype (SEM coated male with same data as holotype but from 4&deg;53.210'S, 73&deg;38.921'W, 7&ndash;10.IX.2017, rainforest, FIT near creek (PER 17-22l ) of Amazonothops aslaki described in Jenkins Shaw et al. (2020) were sent for the permanent deposition to the Museo de Historia Natural de la Universidad Nacional Mayor de San Marcos in Peru (MHN-UNMSM).

In a recent paper (Jenkins Shaw et al. 2020) we stated that the holotype of *Amazonothops aslaki* would be deposited in the Natural History Museum of Denmark (NHMD). According to the conditions of the permit RDG 0328-2017-SERFOR-DGGSPFFS/RDG 356-2017-SERFOR-DGGSPFFS, the holotype and one paratype (SEM coated male with same data as holotype but from 4°53.210'S, 73°38.921'W, 7–10.IX.2017, rainforest, FIT near creek (PER 17-22l) of *Amazonothops aslaki* described in Jenkins Shaw et al. (2020) were sent for the permanent deposition to the Museo de Historia Natural de la Universidad Nacional Mayor de San Marcos in Peru (MHN-UNMSM).
